# Glycine as a potential neuroprotective adjuvant to reduce cisplatin-induced brain inflammation in mice

**DOI:** 10.1186/s12868-026-01023-4

**Published:** 2026-06-28

**Authors:** Hafiz Muhammad Obaid Kazmi, Muhammad Umer Suleman, Syeda Iqra Maqsood, Shahbaz Azam Khan, Iqra Khadam, Saima Mumtaz Khattak, Umer Khalil, Muhammad Mursaleen, Muhammad Mustafeez Waheed Jami, Sumaira Javed, Najeeb Ullah, Muhammad Ikram, Marwan Alqumbaey

**Affiliations:** 1 Ayub Medical College, Abbottabad, Pakistan; 2 Department of Obstetrics & Gynaecology, Training Medical Officer, Ayub Teaching Hospital, Abbottabad, Pakistan; 3 Jhelum College of Nursing and Health Sciences, Jhelum, Pakistan; 4 Federal Medical College, Islamabad, Pakistan; 5 DHQ (WM&DC), Abbottabad, Pakistan; 6 Anatomy Department, Ayub Medical College, Abbottabad, Pakistan; 7 Institute of Basic Medical Sciences, Khyber Medical University, Peshawar, Pakistan; 8 Institute of Pharmaceutical Sciences, Khyber Medical University, Peshawar, Pakistan; 9 Faculty of Medicine and Health Sciences, Sana’a University, Sana’a, Yemen

**Keywords:** Cisplatin, Glycine, Neuroinflammation, Tumor necrosis factor-alpha, Neuroprotection, Hippocampus

## Abstract

**Background:**

Cisplatin-induced neurotoxicity is driven in part by neuroinflammation and oxidative injury in vulnerable brain regions. Glycine has anti-inflammatory and antioxidant properties that may offer neuroprotection against chemotherapy-related brain damage.

**Methods:**

Twenty-five adult male BALB/c mice were randomized into five groups (*n* = 5/group) Group 1 received cisplatin for 14 days; Group 2 received cisplatin plus glycine for 14 days; Group 3 received cisplatin for 28 days; Group 4 received cisplatin for 14 days followed by glycine for 14 days; and Group 5 received cisplatin for 28 days with glycine introduced from day 14 to day 28. Cisplatin was administered intraperitoneally at 3 mg/kg every fourth day, and glycine was given subcutaneously at 1 g/kg daily. The primary outcome was serum TNF-α measured by ELISA. Secondary outcomes were neuronal integrity and optical density in the hippocampus and frontal cortex assessed by Nissl staining. Data were analyzed using one-way ANOVA with Tukey post-hoc testing.

**Results:**

Serum TNF-α levels differed significantly among groups (F = 230.422, *p* < 0.001). Mean TNF-α concentrations were 150.0 pg/mL in Group 1, 130.2 pg/mL in Group 2, 201.4 pg/mL in Group 3, 159.4 pg/mL in Group 4, and 171.0 pg/mL in Group 5. Prolonged cisplatin exposure (Group 3) produced the highest TNF-α levels, whereas concurrent glycine administration during the 14-day regimen (Group 2) resulted in the lowest levels. Compared with the 28-day cisplatin group, both delayed glycine treatment (Group 4) and glycine introduced during the second half of cisplatin exposure (Group 5) were associated with lower TNF-α concentrations. Histological analysis demonstrated reduced Nissl staining intensity and neuronal preservation in cisplatin-only groups, particularly Group 3, whereas glycine-treated groups showed better preservation of neuronal architecture and optical density in the hippocampus and frontal cortex.

**Conclusion:**

Glycine attenuated cisplatin-induced neuroinflammation and preserved neuronal integrity in the hippocampus and frontal cortex of mice. These findings support further preclinical evaluation of glycine as a low-cost adjuvant strategy to reduce chemotherapy-associated neurotoxicity.

**Graphical Abstract:**

**Figure:**
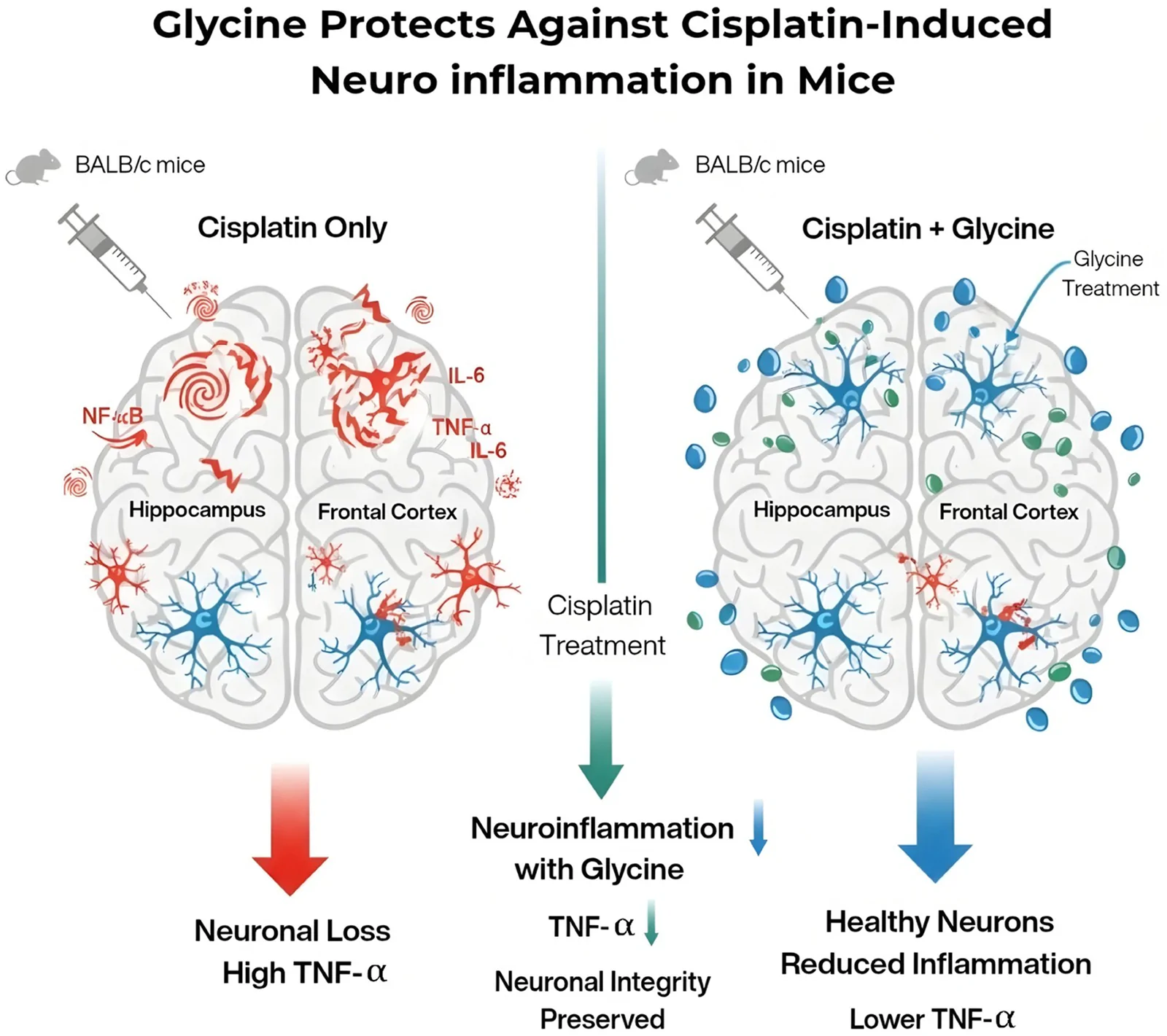

**Supplementary Information:**

The online version contains supplementary material available at https://doi.org/10.1186/s12868-026-01023-4.

## Introduction

 Cisplatin (cis-diamminedichloroplatinum-II) is a widely used chemotherapeutic agent effective against various malignancies, but its clinical utility is limited by severe toxicities including neurotoxicity [1]. In the CNS, cisplatin readily penetrates the blood brain barrier and accumulates in vulnerable regions such as the hippocampus and cortex [2]. Both clinical and animal studies show that cisplatin exposure elevates markers of neuroinflammation in these brain areas [3]. For example, rodent models of cisplatin treatment exhibit dramatically increased hippocampal levels of NF-κB, tumor necrosis factor-α (TNF-α) and interleukin-6 (IL-6), and pro-inflammatory cytokines TNF-α, IL-1β and IL-6 are consistently upregulated in the brain after cisplatin administration [1, 3, 4]. These cytokines are largely produced by activated glia: cisplatin induces microglial and astrocytic activation (as indicated by Iba-1 and GFAP) and drives NF-κB signaling, which in turn upregulates TNF-α, IL-6 and related mediators [5, 6]. Importantly, these inflammatory mediators amplify neuronal injury. For instance, increased TNF-α and IL-6 promote oxidative damage, mitochondrial dysfunction and apoptosis in hippocampal neurons, linking neuroinflammation to the cognitive deficits observed in cisplatin-treated subjects [1, 3].

Mechanistic studies support a vicious cycle of cisplatin toxicity: DNA damage and calcium dysregulation induced by cisplatin trigger excessive reactive oxygen species (ROS) production and lipid peroxidation [1, 3, 7]. The resulting oxidative stress not only activates apoptotic cascades (p53, Bax, caspases) in neurons, but also potentiates pro-inflammatory signaling in glia [1, 3]. Inflammatory transcription factors such as NF-κB become activated by cisplatin, leading to upregulation of TNF-α, IL-1β, IL-6 and other cytokines [1, 3, 8]. Indeed, recent work shows that cisplatin increases NF-κB and its downstream cytokines in hippocampus and cortex, and that astrocytic GFAP and microglial Iba-1 are elevated in this context [1, 9, 10]. In aggregate, cisplatin neurotoxicity appears to be driven by a feed-forward loop in which drug-induced oxidative stress and glial activation collaborate to damage neurons and impair synaptic plasticity [1, 3].

Glycine, the simplest non-essential amino acid has emerged as a potent anti-inflammatory and neuroprotective agent in many settings [11, 12]. Glycine is abundant in plasma and CNS tissue, where it functions as both a neurotransmitter and an immunomodulator [12, 13]. Notably, glycine exerts broad anti-inflammatory effects by inhibiting NF-κB-mediated gene expression and cytokine release [11, 12]. In immune cells such as macrophages and Kupffer cells, glycine decreases endotoxin-induced TNF-α and IL-1β production, and in animal models dietary glycine protects against endotoxin or sepsis-induced organ damage [11, 13, 14]. Mechanistically, glycine activates an inhibitory chloride conductance in inflammatory cells, hyperpolarizing them and blunting Ca²⁺ dependent cytokine release [11]. As a result, glycine supplementation markedly reduces pro-inflammatory cytokines (TNF-α, IL-6, IL-1β) and limits tissue injury in models of shock, reperfusion, and toxic injury [11, 13].

In the nervous system, glycine similarly counteracts inflammatory and oxidative insults. Glycine is a required substrate for glutathione synthesis, and thus supports antioxidant defenses against ROS [13, 15, 16]. It also engages pro-survival signaling: for example, glycine activates PI3K/Akt pathways that inhibit stress kinases and apoptosis [13, 17]. Experimentally, glycine has been shown to protect neurons and glia from insult [18–20]. In a neonatal ethanol model, glycine co-treatment prevented NF-κB activation and dramatically reduced TNF-α and COX-2 expression in the brain, limiting apoptotic neurodegeneration. In stroke models, glycine shifts microglia toward an anti-inflammatory (M2) phenotype by suppressing NF-κB p65 and HIF-1α, thereby reducing infarct size [21]. Similarly, in an aging (D-galactose) mouse model, glycine supplementation reversed the upregulation of hippocampal IL-1β, TNF-α and glial markers (GFAP, Iba-1) caused by D-galactose, concomitant with improvements in synaptic protein expression and cognition [22]. These studies collectively indicate that glycine can dampen CNS inflammation, lowering pro-inflammatory cytokines and oxidative stress while enhancing cell survival signals.

Taken together, the neuroinflammatory cascade elicited by cisplatin (involving NF-κB, TNF-α, IL-6 and ROS) overlaps with pathways modulated by glycine. Given glycine’s documented ability to suppress cytokine production and oxidative damage, we hypothesize that glycine treatment will mitigate cisplatin-induced neuroinflammation. In the present study, we thus investigate the neuroprotective effects of glycine against cisplatin-triggered neuroinflammatory injury in mice. Our objective is to unveil the role of glycine in the reduction of cisplatin-induced neuroinflammation.

## Materials and methods

The article has been prepared in accordance with the ARRIVE guidelines to ensure transparency and rigor in reporting animal research [23].

### Animals

For this investigation, 25 adult male healthy BALB/c mice weighing between 22 and 28 g and 6–8 weeks of age were used. Animals were obtained from NIH, Islamabad. BALB/c mice were selected due to their well-established behavioural and physiological responses, particularly in neurobiological research. Female mice and male mice with disease were excluded because fluctuations in the hormonal cycle during estrous might affect neuroinflammation and cognitive outcomes. Mice were acclimatized for 7 days before experiments and maintained under standard conditions (12 h light/dark cycle, 22 ± 2 °C, humidity 50–60%) with ad libitum access to food and water. A maximum of five mice were housed per cage; cages were enriched with tubes and nesting material.

### Ethics statement

Ethical approval for this study was obtained from the Institutional Animal Care and Use Committee of the Institute of Basic Medical Sciences (IBMS), Khyber Medical University (Approval No. DIRJKMU-AS&RB/GA/002952, dated 16 October 2024). Every procedure complied with national and international ethical standards for animal research, including the Animal Welfare Act (AWA), Public Health Service (PHS) Policy, and adhered to the 3R principles of animal research (Replacement, Reduction, and Refinement) under the approval of the Institutional Animal Care and Use Committee (IACUC).v. Humane killing techniques, frequent behavioural and health monitoring and the use of suitable anesthetics were all part of the efforts to reduce animal suffering.

### Experimental design, group allocation and sample size

A between-group experimental design with five treatment groups (*n* = 5 mice per group; total *N* = 25) was used. Sample size estimation was performed using the Resource-Equation method:

DF = 20 → n = DF/k + 1 = 20/5 + 1 = 5 mice per group.

Mice were allocated by simple random sampling to the following groups:

- **Group 1(G1)**: Cisplatin for 14 days (cisplatin-only short-term toxicity).
- **Group 2(G2)**: Cisplatin plus glycine for 14 days (concurrent protective treatment).
- **Group 3(G3)**: Cisplatin for 28 days (long-term cisplatin toxicity).
- **Group 4(G4)**: Cisplatin for 14 days followed by glycine for 14 days (therapeutic post-treatment).
- **Group 5(G5)**: Cisplatin for 28 days with glycine from day 14 to 28 (delayed/protective during later treatment).

### Drug administration

Cisplatin was administered intraperitoneally at 3 mg/kg every fourth day; dose volumes were adjusted to body weight (approximately 0.1–0.2 mL) using sterile saline as a vehicle. The intended cumulative dose for the 14-day regimen was 12 mg/kg (3 mg/kg × 4 doses). Glycine was administered subcutaneously at 1 g/kg once daily. It was prepared from powder in distilled water and injected into the dorsal scruff (0.1–0.2 mL depending on animal weight). In the supplied protocol, glycine was given 15 min before cisplatin when co-administered. We chose subcutaneous injection as their method because it provided reliable drug absorption while allowing easy repetition of treatments throughout the research period.

All injections were performed using 1-mL insulin syringes (29–31 gauge) under aseptic technique. Animal handling during injections was described as gentle manual restraint; no anaesthetic was reported for injections. Euthanasia for sample collection was performed by cervical dislocation as stated below.

### Sample collection

Blood samples were collected immediately after cervical dislocation for the quantification of pro-inflammatory cytokines. Serum was separated and analyzed using ELISA to measure TNF-α levels. Absorbance values were measured at 450 nm using a microplate reader, and cytokine levels were calculated by reference to the standard curve supplied with the kit. For histological assessment, brain tissues from the frontal cortex and hippocampus were dissected and processed for Nissl staining. The stained sections were imaged, and the optical density was quantified using ImageJ software to evaluate neuronal density and structural integrity.

### ELISA for TNF-α quantification

Following blood collection, samples were allowed to clot and were centrifuged at 3000 rpm for 10 min at 4 °C to isolate serum, which was stored at − 80 °C until analysis. Serum TNF-α was quantified using an ELISA kit (E0117Mo) according to the manufacturer’s protocol. The wells’ absorbance level were measured through the microplates reader after finishing TNF-α quantification using ELISA procedures by setting their wavelength to 450 nm. The analysis plate required several minutes at room temperature to reach equilibrium for a uniform color reaction outcome. The test apparatus detected optical density (OD) readings from all examined samples along with the standards. The unknown TNF-α concentration in samples were determined through comparison of their OD values with the ones derived from the standard curve containing known TNF-α concentration. The experiments were conducted twice or thrice to guarantee both precision and exactness of the results.

### Nissl staining for neuronal integrity

Nissl staining is a widely used histological method to assess neuronal health, where reduced staining intensity reflects neuronal loss and structural damage. To evaluate neuronal integrity in brain regions associated with neuroinflammation, coronal brain sections were stained with cresyl violet (Nissl).Brains were fixed in 4% paraformaldehyde and coronally sectioned on a cryostat or microtome at 5–14 μm thickness. Sections were rehydrated through a graded alcohol series (100% to 70%) and rinsed in distilled water prior to staining.

Sections were stained with 0.5% Cresyl Violet acetate (prepared in distilled water with a few drops of glacial acetic acid) for approximately 10 min at room temperature and briefly rinsed in distilled water to remove excess dye. Dehydration was performed through ascending alcohols (70%, 95%, 100%) for 2–5 min at each step, followed by clearing in xylene for five minutes. Coverslips were mounted using a non-fluorescent mounting medium.

Stained sections from hippocampal subfields (CA1, CA3 and dentate gyrus) and frontal cortex were imaged using a light microscope and high-resolution images were acquired for analysis. Quantitative assessment of optical density and neuronal integrity was performed using ImageJ software; processed values were exported to Microsoft Excel for plotting and statistical analysis.

### Outcome measures and data handling

Primary neuroinflammation outcome: serum TNF-α concentration. Secondary structural outcome: optical density/neuronal density in hippocampus and frontal cortex by Nissl staining. All quantitative image analyses were performed using ImageJ; processed values were exported to Microsoft Excel for graphing; statistical analyses used SPSS.

### Statistical analysis

Data are presented as mean ± SEM. Group comparisons for TNF-α and histological measures were analyzed by one-way ANOVA followed by Tukey’s post-hoc test for pairwise group comparisons. A probability value of *p* < 0.05 was considered statistically significant. Statistical analyses were performed using SPSS version 26 (IBM, USA). Results were visualized through figures, charts, and tables generated using SPSS and Microsoft Excel.

## Results

### ELISA analysis

Serum TNF-α levels were measured in all five experimental groups, and descriptive statistics are presented in Table 1, while individual measurements are provided in Supplementary Table S1. Mean TNF-α concentrations ranged from 130.20 ± 3.11pg/mL in Group 2 to 201.40 ± 6.11pg/mL in Group 3 (mean ± SD), indicating substantial variation among treatment groups. The highest TNF-α levels were observed following prolonged cisplatin exposure, whereas glycine-treated groups demonstrated comparatively lower concentrations.

These findings suggest that cisplatin administration is associated with an increased inflammatory response, while glycine treatment may attenuate this effect. The observed differences in TNF-α levels provided the basis for subsequent inferential analyses using one-way ANOVA (Table 2.1) and Tukey’s post-hoc testing (Table 2.2).

**Table 1:** Descriptive statistics of serum TNF-α levels across experimental groups

Group	*n*	Mean (pg/ml)	SD	SEM
Group 1	5	150.00	4.12311	1.84391
Group 2	5	130.20	3.11448	1.39284
Group 3	5	201.40	6.10737	2.73130
Group 4	5	159.40	2.40832	1.07703
Group 5	5	171.00	2.44949	1.09545

The statistical analysis of TNF-alpha levels across different treatment groups revealed significant differences, as indicated by a one-way ANOVA (Table 2.1). The results demonstrated that prolonged cisplatin exposure (28 days) was associated with elevated TNF-α levels compared with the other experimental groups. Groups treated with glycine, either concurrently or after cisplatin exposure, exhibited lower TNF-alpha levels, suggesting that glycine administration was associated with lower TNF-α levels compared with cisplatin-only treatment.

**Table 2.1:** One-Way ANOVA of serum TNF-α levels among experimental groups

	Sum of Squares	df	Mean Square	F	*p*-value
Between Groups	13972.800	4	3493.200	230.422	< 0.001
Within Groups	303.200	20	15.160		
Total	14276.000	24			

### Post hoc analysis

Post-hoc analysis using Tukey’s test revealed significant differences in TNF-α levels between the experimental groups (Table 2.2). Compared with the cisplatin-only groups, all glycine-treated groups exhibited significantly lower TNF-α concentrations, indicating a reduction in the inflammatory response associated with cisplatin administration. Among the glycine-treated groups, Group 2 demonstrated the lowest TNF-α levels, whereas Groups 4 and 5 also showed reductions relative to the cisplatin-only groups but remained higher than those observed in Group 2. These findings suggest that glycine administration was associated with attenuation of cisplatin-induced inflammation and that the timing of glycine treatment may influence the magnitude of this effect.

**Table 2.2:** Tukey’s Post-hoc analysis of pairwise differences in serum TNF-α levels

Pairwise Comparison	Mean Difference	SEM	*p*-value	95% Confidence Interval (Lower - Upper Bound)
Group 3vs. Group 1	51.40	2.46	< 0.001	44.0312–58.7688
Group 3vs. Group 2	71.20	2.46	< 0.001	63.8312–78.5688
Group 3vs. Group 4	42.00	2.46	< 0.001	34.6312–49.3688
Group 3vs. Group 5	30.40	2.46	< 0.001	23.0312–37.7688
Group 2vs. Group 1	19.80	2.46	< 0.001	12.4312–27.1688
Group 5vs. Group 1	21.00	2.46	< 0.001	13.6312–28.3688
Group 5vs. Group 2	40.80	2.46	< 0.001	33.4312–48.1688
Group 4vs. Group 1	9.40	2.46	0.008	2.0312–16.7688
Group 4vs. Group 2	29.20	2.46	< 0.001	21.8312–36.5688
Group 5vs. Group 4	11.60	2.46	< 0.001	4.2312–18.9688

### Nissl staining results

Nissl-stained sections revealed distinct histological differences among the experimental groups. Groups 1 and 3 showed reduced Nissl staining intensity compared with the glycine-treated groups. These changes were more pronounced following prolonged cisplatin exposure in Group 3, which demonstrated markedly reduced staining intensity and altered neuronal morphology relative to the other experimental groups. In contrast, glycine-treated groups (Groups 2, 4, and 5) demonstrated comparatively preserved staining patterns and neuronal architecture, suggesting attenuation of cisplatin-induced histological damage.

**Fig. 1:**
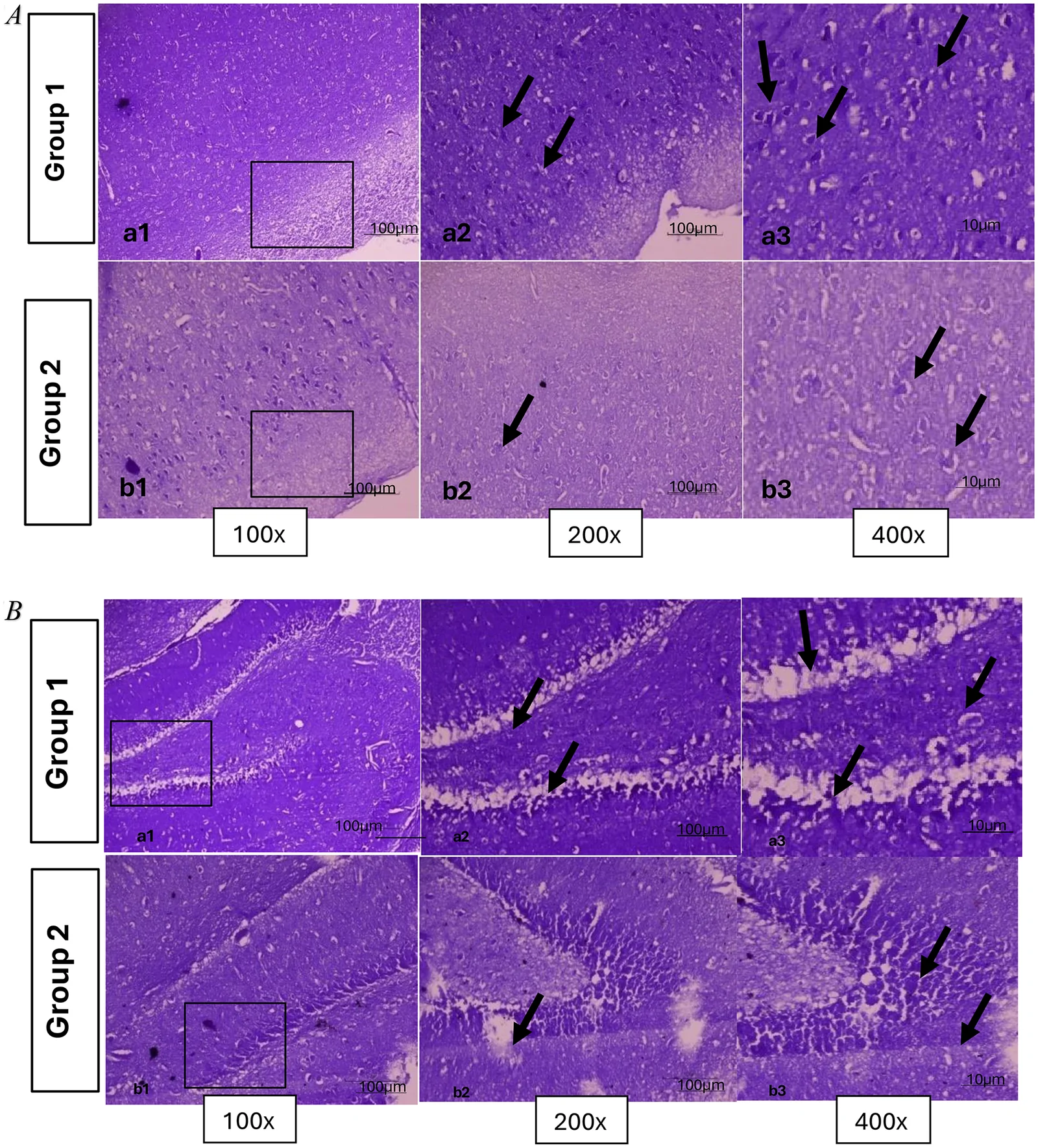

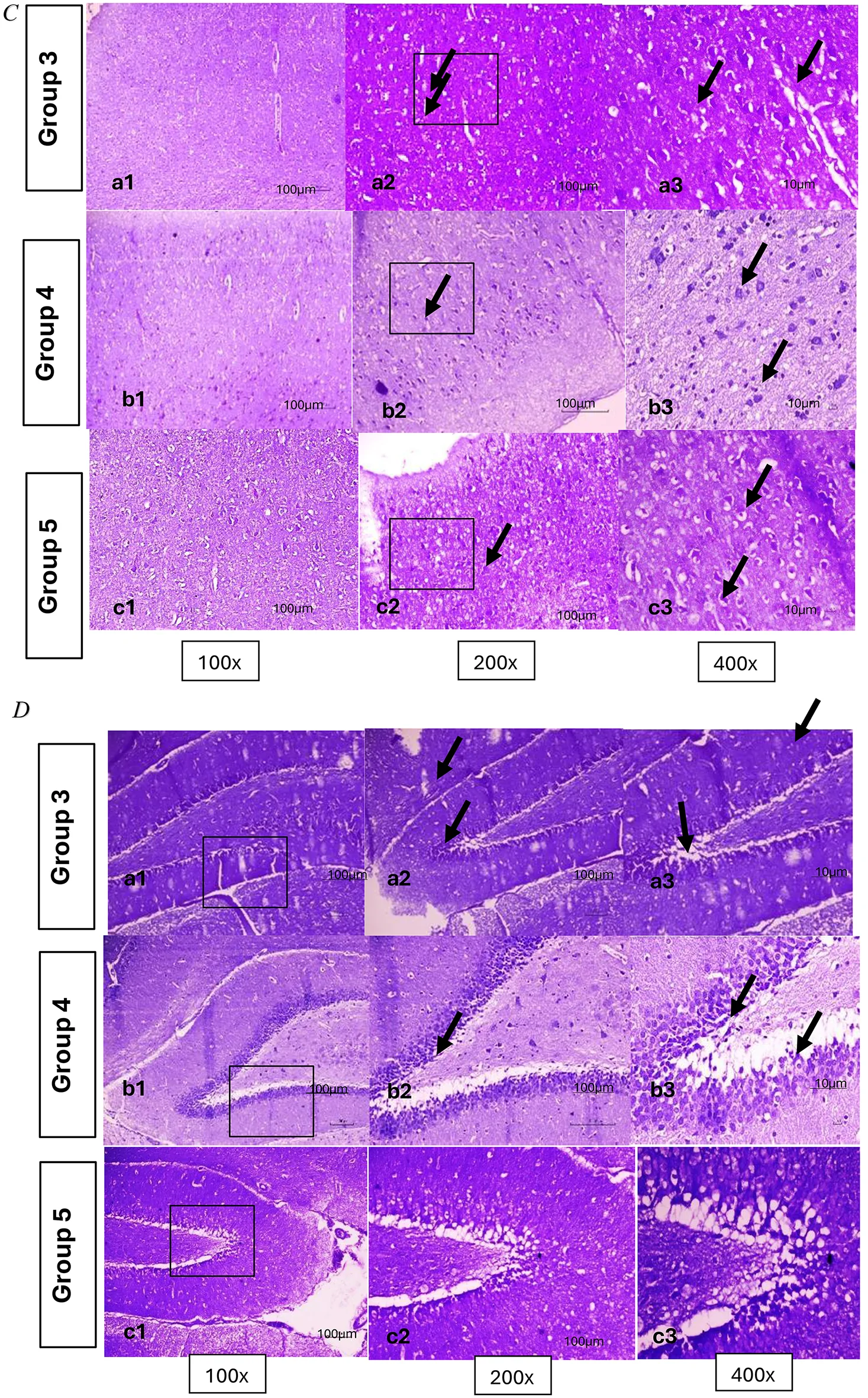
**A**. Nissl Staining in the Frontal Cortex comparison Group 1 (Cisplatin 14 days) and Group 2 (Cisplatin + Glycine for 14 days). Arrow displays positive Nissl staining, 100X (a1, b1), 200X (a2, b2) with scale bar of 100 μm, and 400X (a3, b3) with scale bar of 10 μm. With *n* = 05 in each group. **B**. Nissl Staining in the hippocampus comparison between Group 1 (Cisplatin 14 days) and Group 2 (Cisplatin + Glycine 14 days). Arrow displays positive Nissl staining, 100X (a1, b1), 200X (a2, b2) with scale bar of 100 μm, and 400X (a3, b3) with scale bar of 10 μm. With *n* = 05 in each group. **C**. Nissl Staining in the Frontal Cortex comparison between Group 3, 4 and 5. Arrow displays positive Nissl staining, 100X, (a1, b1, c1), 200X (a2, b2, c2) with scale bar of 100 μm, and 400X (a3, b3, c3) with scale bar of 10 μm. With *n* = 05 in each group. **D**. Nissl Staining in the Hippocampus comparison between Groups 3, 4 and 5. Arrow displays positive Nissl staining, 100X (a1, b1, c1), 200X (a2, b2, c2) with scale bar of 100 μm, and 400X (a3, b3, c3) with scale bar of 10 μm. With *n* = 05 in each group

Representative photomicrographs of Nissl-stained sections from the frontal cortex and hippocampus are presented in Figs. 1A–D. Figure 1A and B compare Group 1 (cisplatin for 14 days) with Group 2 (cisplatin + glycine for 14 days), while Fig. 1C and D compare Groups 3, 4, and 5. Positive Nissl staining is indicated by arrows. Overall, glycine-treated groups exhibited relatively preserved histological appearance compared with the cisplatin-only groups, particularly following prolonged cisplatin exposure.

**Fig. 2:**
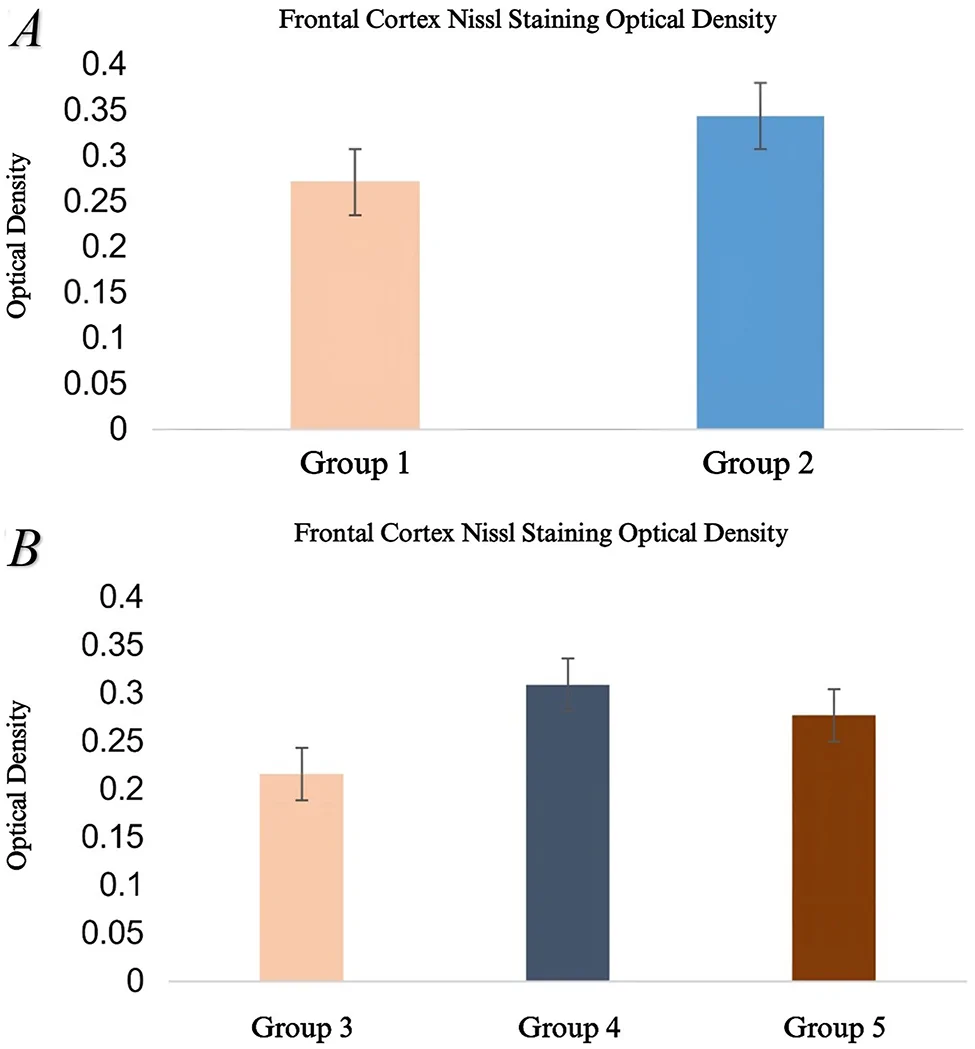

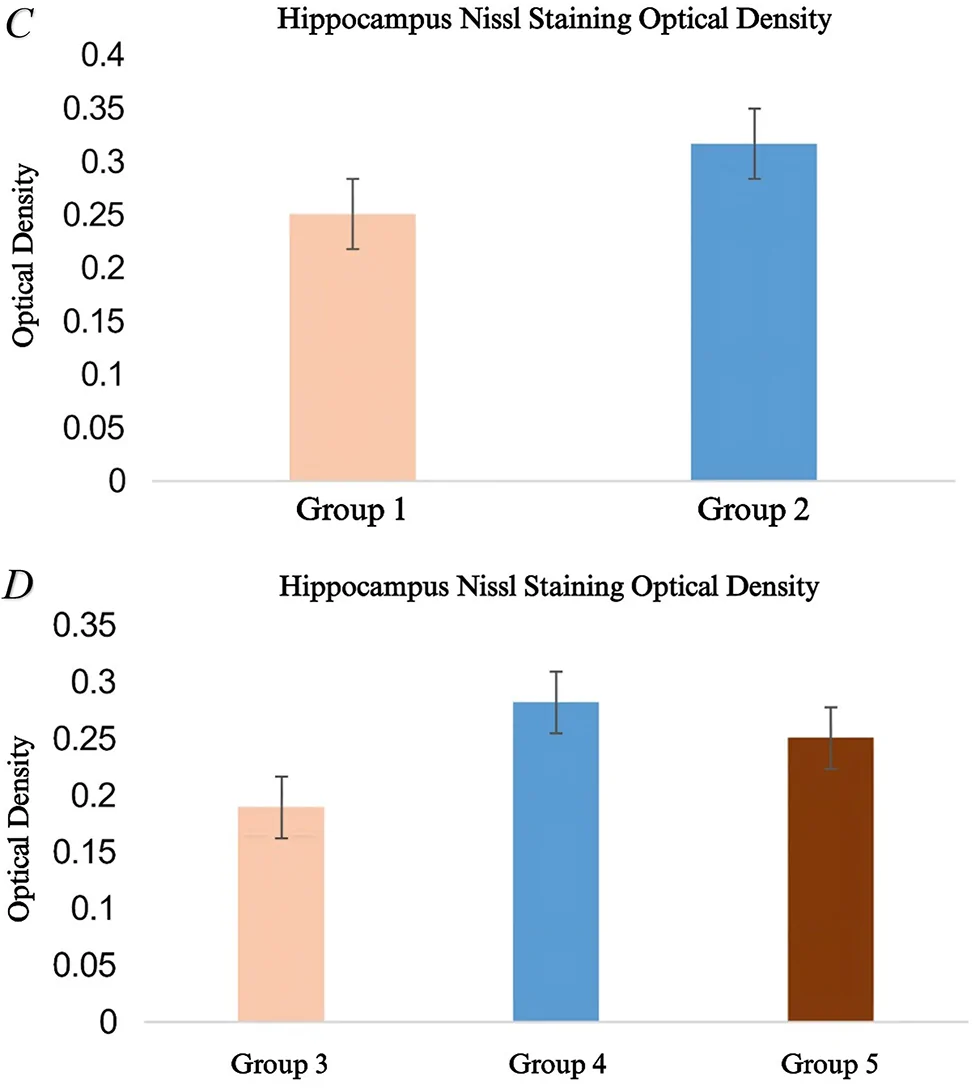
**A**. Optical density of Nissl staining in the frontal cortex of mice treated with cisplatin for 14 days (Group 1) and cisplatin plus glycine for 14 days (Group 2). **B**. Optical density of Nissl staining in the frontal cortex of mice receiving cisplatin for 28 days (Group 3), cisplatin for 14 days followed by glycine for 14 days (Group 4), and cisplatin plus glycine from day 14 to day 28 (Group 5). **C**. Optical density of Nissl staining in the hippocampus of mice treated with cisplatin for 14 days (Group 1) and cisplatin plus glycine for 14 days (Group 2). **D**: Optical density of Nissl staining in the hippocampus of mice receiving cisplatin for 28 days (Group 3), cisplatin for 14 days followed by glycine for 14 days (Group 4), and cisplatin plus glycine from day 14 to day 28 (Group 5)

Optical density measurements were obtained from Nissl-stained sections of the frontal cortex and hippocampus. Individual values are presented in Supplementary Tables S2 and S3, while graphical representations are shown in Figs. 2A–D.

In the frontal cortex, Group 1 showed lower optical density than Group 2, indicating better preservation of Nissl staining in the glycine-treated group (Fig. 2A). Group 3 demonstrated the lowest optical density among the three later-treatment groups, whereas Groups 4 and 5 showed higher optical density values than Group 3, suggesting partial preservation of neuronal staining following glycine administration. Among these two groups, Group 4 exhibited higher optical density than Group 5, indicating that the timing of glycine administration may influence the degree of histological preservation (Fig. 2B).

A similar pattern was observed in the hippocampus. Group 2 showed higher optical density than Group 1 (Fig. 2C), while Group 3 had the lowest optical density among the three later-treatment groups (Fig. 2D). Groups 4 and 5 both demonstrated higher optical density than Group 3, with Group 4 again showing greater preservation than Group 5 as shown in Fig. 2D. These findings indicate that glycine treatment was associated with improved Nissl staining intensity in both brain regions. Higher optical density values were observed in Group 4 than in Group 5, suggesting that the timing of glycine administration may influence the degree of histological preservation.

## Discussion

Our findings suggest that glycine treatment attenuates cisplatin-induced neuroinflammation and preserves neuronal integrity in the hippocampus and frontal cortex of mice. The severe off-target neurotoxicities of established agents like cisplatin underscore the continuous imperative to understand their mechanisms and develop novel strategies to improve therapeutic efficacy [24]. Cisplatin administration was associated with elevated serum TNF-α levels, consistent with previous reports demonstrating inflammatory changes in experimental models of cisplatin-induced neurotoxicity [3, 4]. For instance, Alhowail et al. reported increased hippocampal TNF-α and NF-κB signaling in cisplatin-treated rats [3]. Likewise, Park et al. reported that cisplatin-injected mice exhibited significantly higher hippocampal TNF-α, IL-6 and IL-1β, alongside reduced synaptic protein expression [4].

This elevation of inflammatory mediators is known to mediate synaptic and dendritic damage; cisplatin directly injures hippocampal synapses, causing rapid loss of dendritic spines, a recognized mechanism of cisplatin-induced neurotoxicity [25]. The structural damage aligns with reports that cisplatin impairs synaptic integrity in the prefrontal cortex, resulting in structural and functional alterations in the prefrontal cortex [26]. These studies suggest cisplatin neurotoxicity involves both direct excitotoxic effects and inflammation-driven neuronal injury. This conclusion is supported by evidence that cisplatin crosses the blood–brain barrier, causes persistent disruption, damages mitochondrial function, and triggers oxidative stress and apoptosis [27, 28]. This damage has been associated with mitochondrial dysfunction, altered neuronal homeostasis, and impaired brain function in experimental models, and inhibition of hippocampal cell proliferation and altered apoptotic gene expression [29, 30]. In our study, glycine administration substantially suppressed the cisplatin-induced surge in TNF-α and preserved neuronal morphology, aligning with the growing evidence that controlling neuroinflammation may mitigate cisplatin-induced neurotoxicity. Comparison of the glycine-treated groups suggests that concurrent glycine administration produced the most favourable response in the present study, with lower TNF-α levels and better preservation of Nissl staining intensity than the delayed-treatment groups. In contrast, Groups 4 and 5 also showed improvement relative to cisplatin-only exposure, but the magnitude of protection appeared smaller than that seen with concurrent treatment. These findings suggest that the timing of glycine administration may influence the degree of neuroprotection.

Glycine’s neuroprotective actions are primarily attributed to its well-characterized anti-inflammatory and antioxidant properties [12, 25]. Mechanistically, glycine inhibits NF-κB signaling and subsequent downstream cytokine production across various models [12, 25]. In peripheral immune cells, activation of glycine receptors hyperpolarizes the cell membrane, which reduces Ca2 + dependent inflammatory signaling and decreases the release of TNF-α, IL-6 and IL-1β [12]. In brain-specific contexts, glycine treatment mitigates both astrocyte and microglial activation while lowering pro-inflammatory cytokines. For instance, glycine co-treatment prevented d-galactose-induced astrocytosis and microgliosis in mouse brain, significantly reducing IL-1β and TNF-α [22]. Similarly, in an ethanol neurotoxicity model, glycine inhibited hippocampal NF-κB activation, preventing the elevation of COX-2 and TNF-α and consequently blocking oxidative stress and neuronal apoptosis [17]. Furthermore, in an LPS-induced neuroinflammatory model, glycine demonstrated direct neuroprotection by mitigating neurodegeneration, reducing apoptosis, caspase-3 activity, and TLR4 signaling [31]. In the present study, glycine-treated mice showed lower serum TNF-α levels than cisplatin-only mice, which is consistent with a potential anti-inflammatory effect. Although the specific molecular pathways were not directly assessed experimentally, the present findings are in line with prior literature suggesting that glycine-mediated neuroprotection may involve modulation of inflammatory signaling pathways such as NF-κB. Thus, the reduction in TNF-α observed in this study is compatible with glycine’s established anti-inflammatory profile.

In addition to its anti-cytokine action, glycine has been reported to support neuronal protection by augmenting antioxidant defenses. In the brain, glycine has been associated with enhanced Nrf2/HO-1 activity and reduced reactive oxygen species (ROS), suggesting a role in limiting oxidative injury. Previous studies have shown that glycine can restore antioxidant proteins Nrf2 and HO-1 suppressed by d-galactose and significantly reduce ROS and lipid peroxidation in the mouse hippocampus, suggesting it prevents oxidative damage alongside inhibiting inflammation [22]. This is relevant as cisplatin neurotoxicity involves mitochondrial impairment and ROS generation. This focus on oxidative stress is supported by similar studies where compounds like N-acetylcysteine (NAC) prevent cisplatin-induced neurotoxicity by reducing both inflammation and oxidative damage [32]. In addition, glycine has been linked in the literature to increased cellular glutathione synthesis and improved antioxidant capacity, which may further contribute to its cytoprotective profile [7, 22]. Furthermore, glycine engages pro-survival signaling by activating PI3K/Akt pathways that inhibit stress kinases and apoptosis. The efficacy of targeting these pro-apoptotic stress pathways is supported by research showing that 6-amino flavone attenuates cisplatin-induced neurotoxicity by inhibiting the p-JNK signaling pathway [33]. Glycine itself confers neuroprotection by regulating the c-Jun N-terminal kinase (JNK), a major stress kinase [22]. In our study, the preservation of neuronal morphology in glycine-treated mice is consistent with a combined reduction in inflammatory insult and improved redox balance. Taken together, these findings suggest that glycine may interrupt the cycle of cisplatin-induced oxidative stress and inflammation, although the specific molecular pathways were not directly evaluated in this work [17, 22].

The translational significance of these findings is high, given glycine’s favourable safety profile and pharmacology. As a naturally occurring, non-essential amino acid, glycine is generally well tolerated, even at pharmacological doses. Clinical trials in humans, such as those for schizophrenia or athletic performance, have administered up to 30–60 g/day without reporting major toxicity, apart from mild sedation at very high doses [22]. Our mouse study similarly demonstrated clear neuroprotective benefits with glycine dosing, showing no apparent harm.

Glycine’s wide therapeutic window and low cost strongly support its feasibility for clinical translation. This goal aligns with established critical reviews on maximizing the translatability of cisplatin mouse models to human ‘chemo-brain’ [34]. Glycine supplementation (or formulations that enhance brain glycine, like GlyT1 inhibitors) could be tested as an adjuvant therapy to chemotherapy to prevent cognitive side effects. The regulatory role of glycine transporters (GlyT1 and GlyT2) in the central nervous system is crucial to this translational step [35]. The therapeutic potential of targeting these pathways is further evidenced by a study where a GlyT2 inhibitor provided anti-allodynic and anti-hyperalgesic efficacy in a rat model of cisplatin-induced peripheral neuropathy [36].

In summary, our findings support a model where cisplatin induces neuroinflammation and oxidative stress in the hippocampus and cortex, resulting in neuronal and synaptic damage. Glycine appeared to counter these effects in the current model, as reflected by the reduction in serum TNF-α levels and the preservation of neuronal morphology observed in the glycine-treated groups. These observations are consistent with previous studies suggesting that glycine may modulate inflammatory signaling, including NF-κB-related pathways, and enhance antioxidant defenses [3, 12, 22]. In the present study, lower TNF-α levels were associated with better preservation of neuronal architecture, which parallels earlier reports showing that reduction of TNF-α may be linked to attenuation of cisplatin-induced neurotoxicity [4, 17, 22, 25, 37].This neuroprotective strategy is further validated by research showing that natural antioxidants, such as Vitamin E, successfully ameliorate cisplatin-induced neurotoxicity and improve behavioral outcomes in rats, specifically by targeting oxidative stress [38, 39]. Thus, glycine emerges as a promising candidate neuroprotectant against chemotherapy-induced neurotoxicity.

### Future directions and translational potential

The neuroprotective efficacy of glycine warrants further investigation in clinically oriented models. Future research must assess glycine’s effects on behavioral and cognitive endpoints in chemotherapy-treated animals, utilizing tests like spatial memory or object recognition, to link molecular protection to functional outcomes. Research must also elucidate the involved receptor and signaling pathways (e.g., glycine vs.NMDA receptors). To fully understand glycine’s potential, subsequent research must dissect how it targets the diverse, overlapping pathways underlying neurotoxicity, such as mitochondrial damage, DNA repair disruption, and sustained glial activation, which are comprehensively reviewed as core components of chemo-brain pathogenesis [40]. Optimizing therapy requires detailed dose-response and timing studies; while our data suggests both concurrent and post-treatment regimens are effective, the most practical human schedule needs delineation. Furthermore, exploring the combination of glycine with other protective agents (e.g., antioxidants) could yield additive benefits. Studies should verify that glycine does not compromise cisplatin’s antitumor efficacy. Ultimately, early-phase clinical trials could test whether dietary glycine supplementation ameliorates “chemo-brain” in cancer patients. Glycine’s low cost and safety make it an attractive translational candidate, potentially improving quality of life and reducing long-term neurological morbidity associated with cancer treatment [22, 28].

### Limitations

Despite the fact that this study offers insightful information, a number of limitations must be noted. Firstly, the study was conducted exclusively in young, healthy male BALB/c mice, limiting the generalizability of the findings. In clinical settings, patients receiving cisplatin often represent a heterogeneous population, including elderly individuals, females, and those with comorbidities such as diabetes or systemic inflammation. These factors can significantly influence both susceptibility to neurotoxicity and response to neuroprotective interventions. Future studies should consider incorporating diverse animal models that better reflect patient variability. Additionally, although statistically significant differences were observed between the experimental groups, the relatively small sample size (*n* = 5 per group) may limit the statistical robustness and external validity of the findings. Future studies using larger cohorts are warranted to further validate the observed effects. Another limitation is the absence of a control group without cisplatin exposure. Although previous studies have extensively established cisplatin-induced neuroinflammation and neuronal injury, inclusion of a healthy control group would have provided an additional baseline reference for validating the TNF-α alterations and histopathological changes observed in the present study [1–3]. Furthermore, the present investigation was intentionally focused on biochemical and histopathological endpoints. Although behavioral and cognitive assessments are important for determining the functional relevance of chemotherapy-induced neurotoxicity, these outcomes were beyond the scope of the current article. Future studies integrating molecular, structural, and behavioral assessments would provide a more comprehensive understanding of the translational significance of glycine-mediated neuroprotection. Finally, the absence of a post-treatment follow-up period limits our ability to assess the long-term durability of glycine’s protective effects. Whether the reductions in neuroinflammation and neuronal damage observed persist after treatment cessation remains an open question. Longitudinal studies are needed to determine whether glycine confers transient relief or lasting neuroprotection.

## Conclusion

The findings of this study underscore the potential of glycine as a neuroprotective adjuvant against cisplatin-induced neurotoxicity. Glycine administration was associated with lower serum TNF-α levels and better preservation of neuronal morphology in the hippocampus and frontal cortex. Further studies are warranted to elucidate the underlying mechanisms and to evaluate the translational potential of glycine for mitigating chemotherapy-associated neurotoxicity.

## Supplementary Information

Below is the link to the electronic supplementary material.

Supplementary Material 1

## Data Availability

All data supporting the findings of this study are included within the main document.

## Abbreviations

TNF-α: Tumor necrosis factor-alpha
IL-6: Interleukin-6
IL-1β: Interleukin-1 beta
NF-κB: Nuclear factor kappa-light-chain-enhancer of activated B cells
ROS: Reactive oxygen species
COX-2: Cyclooxygenase-2
GFAP: Glial fibrillary acidic protein
Iba-1: Ionized calcium-binding adapter molecule 1
PI3K/Akt: Phosphoinositide 3-kinase / Protein kinase B (Akt)
Nrf2: Nuclear factor erythroid 2–related factor 2
HO-1: Heme oxygenase-1
ELISA: Enzyme-linked immunosorbent assay
OD: Optical density
ImageJ: Image analysis software
SPSS: Statistical Package for the Social Sciences
ANOVA: Analysis of variance
SEM: Standard error of the mean
IACUC: Institutional Animal Care and Use Committee
ARRIVE: Animal Research: Reporting of In Vivo Experiments
AWA: Animal Welfare Act
PHS: Public Health Service
3Rs: Replacement, Reduction, Refinement
NIH: National Institute of Health (as cited in the manuscript: NIH, Islamabad)
IBMS: Institute of Basic Medical Sciences
IP: Intraperitoneal
SC: Subcutaneous
CA1/CA3: Cornu Ammonis regions 1 and 3
DG: Dentate gyrus
NAC: N-acetylcysteine
LPS: Lipopolysaccharide
TLR4: Toll-like receptor 4
JNK: c-Jun N-terminal kinase
µm: Micrometer
