# Supplementary material for: Glycine as a potential neuroprotective adjuvant to reduce cisplatin-induced brain inflammation in mice
Source: BMC Neurosci. 2026 Jun 28;27:34. doi: 10.1186/s12868-026-01023-4 (PMC13573425; doi:10.1186/s12868-026-01023-4)
Supplement: Supplementary file 1 [file 12868_2026_1023_MOESM1_ESM.docx]

Supplementary Table S1. TNF-alpha Levels in Different Treatment Groups

| Group | Sample Well ID | Value (pg/ml) |
| --- | --- | --- |
| Group 1 (Cisplatin for 14 days) | A2 | 150 |
| Group 1 (Cisplatin for 14 days) | B2 | 155 |
| Group 1 (Cisplatin for 14 days) | C2 | 145 |
| Group 2 (Cisplatin + Glycine) | D2 | 130 |
| Group 2 (Cisplatin + Glycine) | E2 | 135 |
| Group 2 (Cisplatin + Glycine) | F2 | 128 |
| Group 3 (Cisplatin for 28 days) | G2 | 200 |
| Group 3 (Cisplatin for 28 days) | G3 | 195 |
| Group 3 (Cisplatin for 28 days) | G4 | 210 |
| Group 4 (Cisplatin then Glycine) | A3 | 160 |
| Group 4 (Cisplatin then Glycine) | B3 | 162 |
| Group 4 (Cisplatin then Glycine) | C3 | 158 |
| Group 5 (Cisplatin + Glycine) | H2 | 170 |
| Group 5 (Cisplatin + Glycine) | H3 | 168 |
| Group 5 (Cisplatin + Glycine) | H4 | 172 |

| *Supplementary Table S2. Optical densities of the frontal cortex among different groups.* | | | | | | | | |
| --- | --- | --- | --- | --- | --- | --- | --- | --- |
| Optical density of Nissl-stained slides at 20x | | | | | | | | |
|  |  |  |  |  |  |  |  |  |
| Group 1: Cisplatin for 14 Days (Moderate Neuroinflammation) | | | | | | | | |
|  | | | | | | | | |
| Mouse 1 | | Mouse 2 | | Mouse 3 | | Mouse 4 | | Mouse 5 |
| Frontal Cortex | | Frontal Cortex | | Frontal Cortex | | Frontal Cortex | | Frontal Cortex |
| 0.27518456 | | 0.28123467 | | 0.27314568 | | 0.27924568 | | 0.27713458 |
| 0.26914578 | | 0.27519458 | | 0.26713457 | | 0.27312456 | | 0.27112458 |
| 0.26314894 | | 0.26914578 | | 0.26112569 | | 0.26713478 | | 0.26513478 |
| 0.26916 |  | 0.275192 |  | 0.267135 |  | 0.273168 |  | 0.271131 |
| Group 2: Cisplatin + Glycine for 14 Days (Partial Neuroprotection) | | | | | | | | |
|  | | | | | | | | |
| Mouse 1 | | Mouse 2 | | Mouse 3 | | Mouse 4 | | Mouse 5 |
| Frontal Cortex | | Frontal Cortex | | Frontal Cortex | | Frontal Cortex | | Frontal Cortex |
| 0.34812456 | | 0.35614578 | | 0.34512458 | | 0.35212458 | | 0.35012457 |
| 0.34124567 | | 0.34912458 | | 0.33814578 | | 0.34514578 | | 0.34314567 |
| 0.33512458 | | 0.34312457 | | 0.33212456 | | 0.33912458 | | 0.33712456 |
| 0.341498 |  | 0.349465 |  | 0.338465 |  | 0.345465 |  | 0.343465 |
|  |  |  |  |  |  |  |  |  |
| Group 3: Cisplatin for 28 Days (Severe Neuroinflammation) | | | | | | | | |
|  | | | | | | | | |
| Mouse 1 | | Mouse 2 | | Mouse 3 | | Mouse 4 | | Mouse 5 |
| Frontal Cortex | | Frontal Cortex | | Frontal Cortex | | Frontal Cortex | | Frontal Cortex |
| 0.21814578 | | 0.22514568 | | 0.22013457 | | 0.22814567 | | 0.22313456 |
| 0.21113467 | | 0.21812456 | | 0.21312456 | | 0.22112456 | | 0.21612457 |
| 0.20512456 | | 0.21212456 | | 0.20712458 | | 0.21512458 | | 0.21012458 |
| 0.211468 |  | 0.218465 |  | 0.213461 |  | 0.221465 |  | 0.216461 |
| Group 4: Cisplatin for 14 Days, Then Glycine for 14 Days (Improved Neuroprotection) | | | | | | | | |
|  | | | | | | | | |
| Mouse 1 | | Mouse 2 | | Mouse 3 | | Mouse 4 | | Mouse 5 |
| Frontal Cortex | | Frontal Cortex | | Frontal Cortex | | Frontal Cortex | | Frontal Cortex |
| 0.31212457 | | 0.32113457 | | 0.31012457 | | 0.31913457 | | 0.31712456 |
| 0.30514568 | | 0.31414578 | | 0.30314568 | | 0.31214568 | | 0.31014568 |
| 0.29912456 | | 0.30812457 | | 0.29712458 | | 0.30612457 | | 0.30412458 |
| 0.305465 |  | 0.314468 |  | 0.303465 |  | 0.312468 |  | 0.310465 |
| Group 5: Cisplatin for 28 Days, Glycine Started on the 14th Day for 14 Days (Delayed  Neuroprotection) | | | | | | | | |
|  | | | | | | | | |
| Mouse 1 | | Mouse 2 | | Mouse 3 | | Mouse 4 | | Mouse 5 |
| Frontal Cortex | | Frontal Cortex | | Frontal Cortex | | Frontal Cortex | | Frontal Cortex |
| 0.28112457 | | 0.28914568 | | 0.27612457 | | 0.28714568 | | 0.28312457 |
| 0.27514568 | | 0.28312457 | | 0.27014568 | | 0.28112457 | | 0.27714568 |
| 0.26912458 | | 0.27712459 | | 0.26412459 | | 0.27512458 | | 0.27112459 |
| 0.275132 |  | 0.283132 |  | 0.270132 |  | 0.281132 |  | 0.277132 |

| *Supplementary Table S3. showing optical densities of hippocampus among different groups* | | | | | | | | |
| --- | --- | --- | --- | --- | --- | --- | --- | --- |
| Optical density of Nissl stained slides at 20x | | | | | | | | |
| Group 1: Cisplatin for 14 Days (Moderate Neuroinflammation) | | | | | | | | |
|  | | | | | | | | |
| Mouse 1 | | Mouse 2 | | Mouse 3 | | Mouse 4 | | Mouse 5 |
| Hippocampus | | Hippocampus | | Hippocampus | | Hippocampus | | Hippocampus |
| 0.25516789 | | 0.26112458 | | 0.25314578 | | 0.25913478 | | 0.25713478 |
| 0.24914567 | | 0.25516789 | | 0.24712458 | | 0.25314568 | | 0.25114568 |
| 0.24312456 | | 0.24914578 | | 0.24112457 | | 0.24712459 | | 0.24512458 |
| 0.249146 |  | 0.255146 |  | 0.247132 |  | 0.253135 |  | 0.251135 |
| Group 2: Cisplatin + Glycine for 14 Days (Partial Neuroprotection) | | | | | | | | |
|  | | | | | | | | |
| Mouse 1 | | Mouse 2 | | Mouse 3 | | Mouse 4 | | Mouse 5 |
| Hippocampus | | Hippocampus | | Hippocampus | | Hippocampus | | Hippocampus |
| 0.32114567 | | 0.32912458 | | 0.31814568 | | 0.32514578 | | 0.32314568 |
| 0.31512458 | | 0.32314567 | | 0.31212458 | | 0.31912458 | | 0.31712456 |
| 0.30912457 | | 0.31712459 | | 0.30612457 | | 0.31312459 | | 0.31112457 |
| 0.315132 |  | 0.323132 |  | 0.312132 |  | 0.319132 |  | 0.317132 |
|  |  |  |  |  |  |  |  |  |
| Group 3: Cisplatin for 28 Days (Severe Neuroinflammation) | | | | | | | | |
|  | | | | | | | | |
| Mouse 1 | | Mouse 2 | | Mouse 3 | | Mouse 4 | | Mouse 5 |
| Hippocampus | | Hippocampus | | Hippocampus | | Hippocampus | | Hippocampus |
| 0.19113456 | | 0.19812457 | | 0.19312456 | | 0.20112458 | | 0.19612458 |
| 0.18512457 | | 0.19214568 | | 0.18714568 | | 0.19512456 | | 0.19012456 |
| 0.17912458 | | 0.18612458 | | 0.18112459 | | 0.18912457 | | 0.18412457 |
| 0.185128 |  | 0.192132 |  | 0.187132 |  | 0.195125 |  | 0.190125 |
| Group 4: Cisplatin for 14 Days, Then Glycine for 14 Days (Improved Neuroprotection) | | | | | | | | |
|  | | | | | | | | |
| Mouse 1 | | Mouse 2 | | Mouse 3 | | Mouse 4 | | Mouse 5 |
| Hippocampus | | Hippocampus | | Hippocampus | | Hippocampus | | Hippocampus |
| 0.28512457 | | 0.29414568 | | 0.28314567 | | 0.29214568 | | 0.29012458 |
| 0.27914568 | | 0.28812457 | | 0.27712456 | | 0.28612457 | | 0.28412456 |
| 0.27312459 | | 0.28212456 | | 0.27112458 | | 0.28012458 | | 0.27812459 |
| 0.279132 |  | 0.288132 |  | 0.277132 |  | 0.286132 |  | 0.284125 |
| Group 5: Cisplatin for 28 Days, Glycine Started on the 14th Day for 14 Days (Delayed Neuroprotection) | | | | | | | | |
|  | | | | | | | | |
| Mouse 1 | | Mouse 2 | | Mouse 3 | | Mouse 4 | | Mouse 5 |
| Hippocampus | | Hippocampus | | Hippocampus | | Hippocampus | | Hippocampus |
| 0.25514567 | | 0.26312458 | | 0.25014568 | | 0.26112457 | | 0.25714568 |
| 0.24912458 | | 0.25714568 | | 0.24412458 | | 0.25512458 | | 0.25112457 |
| 0.24312459 | | 0.25112457 | | 0.23812457 | | 0.24912456 | | 0.24512458 |
| 0.249132 |  | 0.257132 |  | 0.244132 |  | 0.255125 |  | 0.251132 |
